# A Comparison Study of the Blood Component Quality of Whole Blood Held Overnight at 4**°**C or Room Temperature

**DOI:** 10.1155/2013/523539

**Published:** 2013-09-05

**Authors:** Shichun Wang, Tiantian Wang, Yahan Fan, Shan Huang, Zhongmei Yi, Ruiqing Li, Shuming Zhao

**Affiliations:** Department of Blood Transfusion, Southwest Hospital, The Third Military Medical University, No. 30 Street Gaotanyan, Chongqing 400038, China

## Abstract

*Background*. The use of plasma frozen within 24 hrs is likely to increase. Whole blood (WB) and buffy coats (BCs) can be held for a few hrs or overnight before processing. *Methods*. Twenty-four bags of WB for plasma and 12 bags for platelet (PLT) concentrates were collected. The fresh frozen plasma (FFP) was prepared within 6 hrs. I-FP24 and II-FP24 samples were prepared either from leukodepleted WB that was held overnight or from WB that was held overnight before leukodepletion. The PLT concentrates (PCs) were prepared from BCs within 6 hrs (PC1) and within 18 to 24 hrs (PC2). The typical coagulation factors and some biochemical parameters were determined. *Results*. Compared to the FFP samples, the levels of FVII and FVIII in the I-FP24 and II-FP24 samples decreased significantly. The pH, Na^+^, LDH, and FHb levels differed significantly between II-FP24 and FFP. Compared to PC1, PC2 exhibited lower pH, pO_2_, and Na^+^ levels, a higher PLT count, and increased pCO_2_, K^+^, Lac, and CD62P expression levels. *Conclusion*. FP24 is best prepared from WB that was stored overnight at 4°C and then leukodepleted and separated within 24 hrs. PCs are best produced from BCs derived from WB that was held overnight at room temperature.

## 1. Introduction 

An important step in safeguarding the quality and safety of the blood supply is recruiting volunteer donors from low-risk populations and producing qualified blood and blood components. Volunteer donor recruitment is a challenging proposition worldwide. In China, it was particularly difficult before 1998. Traditional Chinese culture believes that the loss of even a small amount of blood has a substantial detrimental health effect. Some people also believe that blood donation is a disloyal action against one's ancestors. Old cultural beliefs, combined with inadequate efforts to mobilize volunteer donors, have led to a chronic shortage of blood products in some areas of China [1]. Therefore, many blood centers have tried to prepare more blood components, such as platelet (PLT) concentrates (PCs), from whole blood (WB) to meet the need for blood products.

WB units are generally held at ambient temperatures (20–24°C) when PCs will be prepared within 6 hrs of the blood collection. The United States Food and Drug Administration (FDA) guidelines during the early 1980s allowed the preparation of components at 20 to 24°C within 8 hrs of collection and a 5-day storage period for PCs. The impetus for extending the time to 8 hrs reflected an increased production demand for PCs and the limitations of the 6 hr period with respect to the collection of a large percentage of WB units at mobile drives, which were sometimes quite distant from the component production laboratories. Currently, because of the overnight holding practice, component manufacturing, including PLT preparation, can be logistically facilitated with related cost savings. Many countries have converted to overnight-hold blood processing because of these advantages [2]. Recently, the Canadian Blood Services has adopted the overnight hold approach to prepare PCs from buffy coats [3].

Currently, the most commonly used plasma product is fresh frozen plasma (FFP), which can be obtained from a plasmapheresis collection or donated WB. FFP must be prepared and frozen within 6 or 8 hrs from the time of collection, according to the usage of different anticoagulants for WB storage. These requirements for FFP preparation could limit the number of WB units that can be processed into FFP because of the blood collections from blood-collecting vehicles. WB stored overnight at 4°C can be used to manufacture plasma and concentrated red blood cells but not for PCs because of the deleterious effect of 4°C storage on PLT quality. PLTs do not tolerate refrigeration and disappear rapidly from circulation if subjected to chilling before transplantation [4–6]. Thus, WB used for PC separation must be stored at room temperature. Currently, PC preparation according to the BC removal method is widely practiced [7–9] and is associated with significantly better PLT recoveries and less PLT activation immediately after preparation [10]. However, it is also inexpedient for PCs that are prepared during the immediate processing of fresh WB. In addition, an overnight hold of BC is good for the PC quantity and quality [11]. There are numerous operational and economic advantages to blood services from ambient WB storage within 24 hrs because of the reduced restrictions on blood processing for PC separation within 8 hrs of collection.

The aim of this study was to compare the quality, as measured by the coagulation factor levels, of plasma prepared from WB that was processed within 8 hrs after collection and storage at 4°C, within 18–24 hrs of storage at 4°C, or within 18–24 hrs of storage at ambient temperature, and the quality of PCs from the BCs processed either from fresh WB or from WB that was held overnight at room temperature.

## 2. Materials and Methods

### 2.1. Study Design

**Figure 1:**
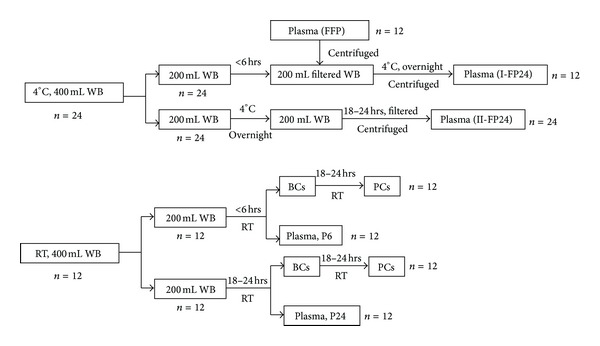
Experimental algorithm for the plasma and PCs production validation with WB held overnight (*n* = 24/12 units in each arm). The plasma was frozen as soon as possible after preparation.

The blood component quality of WB that was stored overnight was evaluated; the frame paragraph of these experiments is shown in Figure 1. Briefly, 400 mL WB samples were collected from normal volunteer donors according to standard procedures in the blood donation law of the People's Republic of China (24 bags used for plasma preparation were stored at 4°C, and 12 bags used for PCs preparation were stored at room temperature). The study protocol was approved by the Southwest Hospital Human Research and Ethics Committee. Each WB sample (400 mL ± 10%) was collected into blood collection packs that contained a CPD anticoagulant (Nigale Biomedical Co., Ltd., Sichuan, China), and then equal volumes were divided between 2 units (200 mL/unit). The blood components were processed in a 2-step centrifugation protocol (2,600 rpm or 1,642 ×g for 15 min, followed by 1,000 rpm or 263 ×g for 6 min in a J6-MC centrifuge; Beckman, Miami, FL, USA). The plasma was removed, and the packed RBCs were resuspended in one of the additive solutions SAG-M (100 mL) (Nigale Biomedical Co., Ltd, Sichuan, China). The FFP was prepared within 6 hrs from 1/2 (*n* = 12) of the leukodepleted WB with a WB in-line leukocyte blood filter (Nigale Biomedical Co., Ltd., Sichuan, China). The I-FP24 (I-FP24, 6 hrs/18–24 hrs) were prepared from leukodepleted blood held overnight (*n* = 12) using a WB in-line leukocyte blood filter (Nigale Biomedical Co., Ltd, Sichuan, China) from WB within 6 hrs, or II-FP24 (II-FP24, 18–24 hrs/18–24 hrs) from WB held overnight and then leukodepleted using a WB in-line leukocyte blood filter (Nigale Biomedical Co., Ltd, Sichuan, China) (*n* = 24). One batch of PCs was prepared from BCs (stored overnight) obtained from fresh WB (fresh/stored, *n* = 12, control group, PC1), and the other batch of PCs was prepared from BCs obtained from WB that was stored overnight at room temperature (stored/fresh, *n* = 12, experimental group, PC2). At the same time, plasma from the 2 groups was prepared, including P6 (*n* = 12) from the PC1 group and P24 (*n* = 12) from the PC2 group. All plasma samples were stored in a −30°C freezer before testing, and all PCs were tested after separation. The typical coagulation factors (FV, FVII, FVIII, Fib) and some biochemical indicators (K^+^, Na^+^, Cl^−^, TP, lactic dehydrogenase [LDH], glucose [GLU], FHb, pH) were observed in all plasma samples. The PLT count and the lactate, pCO_2_, pO_2_, HSR, pH, GLU, and CD62P expression levels were measured in all PCs.

### 2.2. In Vitro Assays

In general, the following methods were used. 

#### 2.2.1. WB Collection and Processing

WB was collected in disposable plastic blood bag **(**110414, Nigale Biomedical Co., Ltd, Sichuan, China) and then refrigerated in a blood refrigerator (HXC-158, Haier, Shanghai, China) or held at room temperature. The plasma from WB which was held at 4°C was prepared by a 2-step centrifugation (3500 rpm or 3128 ×g for 10 min, J6-MC, Beckman, Miami, USA), frozen in freezer (MDF-U538, Sanyo, Osaka, Japan), thawed in plasma thawer (KJX III, Szmic, Suzhou, China) at 37°C. PCs were prepared by a 2-step centrifugation (2600 r.p.m. or 1642 ×g for 15 min followed by 1000 r.p.m. or 263 ×g for 6 min, J6-MC, Beckman, Miami, USA). 

#### 2.2.2. Coagulation Factors

The clotting factor activity levels were determined with an automatic blood coagulation analyzer (CA1500, Sysmex, Kobe, Japan), and the assay reagents included coagulation factors Fib, FVII, FVIII, APTT, and PT (537991, 500725A, 546529A, 537485A, 545363, Dade Behring Marburg, Marburg, Germany) and coagulation factors FV (503587A, Chengdu Xiehe Biotechnology Co., Ltd., Chengdu, China). 

#### 2.2.3. Biochemical Parameters

The glucose and LDH levels were measured using dry chemistry on a chemistry analyzer (AU2700, Olympus, Shimadzu Tokyo, Japan). The FHb level was measured by a 3-wavelength method (380 nm, 415 nm, 450 nm) on a microplate reader (1500-992, Thermo Fisher Scientific, Waltham, MA, USA). The Tris-HCl solution (62.4 mmol/L, pH 8.0) was prepared as the reagent. Briefly, the instrument was zeroed, and the plasma sample was diluted to a 3 mL as Tris-HCl solution : plasma = 9 : 1 (2700  *μ*L : 300 *μ*L) and measured the absorbance against the Tris-HCl solution as blank. The level of FHb is calculated as the formula: FHb (mg/L) = [1.68 × *A*_415_ − 0.84 (*A*_380_ + *A*_450_)] × 1000 mg/L.

#### 2.2.4. Quantity and Quality of PCs

The PLT counts were obtained using an automated cell analyzer (KX-21N, Sysmex, Kobe, Japan). The pH, pO_2_, pCO_2_, glucose, and lactate levels were measured with a blood gas analyzer (ABL715, Radiometer, Copenhagen, Denmark), or pH was measured with a pH meter (PB-21, Sartorius AG, Goettingen, Germany). The glucose and LDH levels were measured using dry chemistry with a chemistry analyzer (AU2700, Olympus, Shimadzu, Tokyo, Japan). The level of HSR and the PAgT were measured with a spectrophotometer (7200, Unic, Bern, Switzerland) and an adhesion meter (HY-I, Beijing Hongrunda instrument Co., Ltd, Beijing, China). The PLT surface CD62P expression was measured with flow cytometry (FACSCalibur, Beckman Coulter, Miami, FL) and fluorescein isothiocyanate (FITC) or phycoerythrin-labeled monoclonal antibodies (CD41 FITC (Lot: 555469), CD62-P PE (Lot: 348107), and Mouse IgG-1 PE (Lot: 555748), BD, USA).

### 2.3. Statistical Analysis

All test unit groups were compared with their respective reference groups. The results are expressed as the mean ± standard deviation (SD) and the median (range). Data were analyzed with the SPSS software program (version 13.0, SPSS Inc., Chicago, IL, USA). Comparisons were performed with a two-sample *t*-test with a 95% confidence interval (CI) or a one-way analysis of variance (ANOVA) and Dunnett's test to identify the differences between each storage method. A *P* value < 0.05 was considered to be significant.

## 3. Results

### 3.1. Plasma Appearance Observations

All frozen plasma samples were warmed to 37°C in a water bath on the third day after collection, and no precipitation emerged.

### 3.2. Effect of Overnight Holding on In Vitro Plasma Quality

The clotting factor analysis of plasma processed under various conditions is shown in Table 1. The plasma was processed approximately 6 hrs after collection or at 18–24 hrs in groups I-FP24 and II-FP24. Compared to FFP, I-FP24 decreased by 22.6%, 29.38%, 32.89%, and 40.71%, including significantly lower FV, FVII, and FVIII levels (*P* < 0.05); in II-FP24, the decreases were 1.9%, 12.60%, 14.91%, and 32.86%, including significantly lower FVIII levels (*P* < 0.05). Compared to FFP and I-FP24, II-FP24 had higher levels of K^+^, LDH, and FHb. The effect of overnight holding of WB at room temperature on the in vitro plasma quality was also analyzed. Compared to P6, P24 had higher levels of Na^+^, K^+^, and FHb and lower levels of Cl^−^, pH, and FVIII. Differences in the pH, Na^+^, LDH, and FHb levels were observed between the II-FP24 and P24 samples.

### 3.3. Effect of Overnight Holding of WB at Room Temperature on In Vitro PC Quality

All PCs had PLT counts well above the current acceptance level of 2.0 × 10^10^/U. Compared to the control group, PC2 had a higher PLT count (3.49 ± 1.19 × 10^10^/U versus 2.78 ± 1.49 × 10^10^/U), which was 25.54% higher than that of PC1 and 74.50% higher than that of the current acceptance level. The PLT in vitro functional parameters were not significantly different, but differences were observed with respect to CD62P expression (11.93 ± 0.18 versus 10.06 ± 0.28, *P* < 0.05) and some biochemical indicators (lower pH, pO_2_ and Na^+^ levels and higher pCO_2_, K^+^, and Lac levels; Table 2).

## 4. Discussion

Blood collection, processing, and storage conditions significantly influence the quality of blood components, and consequently, these processes are closely regulated to maximize safety and efficacy. The current FDA regulations require that WB units held at room temperature must be processed within 8 hrs after collection, or alternatively, the WB units must be refrigerated and processed within 24 hrs [12]. The Council of Europe (COE) guidelines specify that WB units can be held for up to 24 hrs at room temperature provided that the units are rapidly cooled to 20–22°C immediately after collection. The Chinese guidelines require that the WB units are stored at 4°C as soon as possible after collection and are processed within 8 hrs. Overnight room temperature WB holding has both logistical advantages and some disadvantages that potentially affect the quality of components, particularly red blood cells (RBCs). In recent years, Chinese blood centers have tried to improve the national blood supply and safety. In China, WB units for clinical use are collected at blood centers. More than 400 blood centers are operated at 3 levels: provincial, regional, and county. Local government health offices oversee blood center operations. The WB unit donation models in China have changed from paid donors before 1998 to employer-organized donors after the new blood donation law took effect in 1998, and in most areas of China, donors are now volunteers [1]. The blood donation law bans all paid WB donations for clinical use and encourages all Chinese citizens between the ages of 18 and 55 years who meet the health criteria for blood donation to donate blood voluntarily. According to the law, WB units are mostly collected at blood centers or mobile street stations in strategic locations that are run by blood centers. One or 2 units (200 or 400 mL) of WB can be drawn from 1 volunteer, stored in a refrigerator and transported at 4°C by a blood vehicle, and separated into blood components at 4°C within 8 hrs. Currently, most blood centers report that they have already achieved the goal of meeting all of their region's blood needs with volunteer donations.

In early 1999, O'Neill et al. studied the quality of FP24 by measuring clotting factors in plasma obtained from CPD WB after storage at 22°C and 4°C for as long as 24 hrs before frozen storage at −18°C for 1 month. The authors confirmed that plasma separated from WB that had been stored for up to 24 hrs after collection could be used under all conditions that required FFP for transfusion [13]. In 2005, Cardigan et al. also examined the quality of FFP produced from WB stored at 4°C overnight [14]. The authors suggested that there was good retention of relevant coagulation factor activity in plasma produced from WB that had been stored at 4°C for 18 to 24 hrs and that this would be an acceptable product for most patients who required FFP. Several other studies have assessed the stability of FP24 when thawed and stored at 4°C for up to 5 days [15]. The studies discovered that FFP and FP24 contained adequate coagulation factor activities to maintain hemostatic activity. In China, the guideline requires that the FVIII level in >75% of FFP must be >0.7 IU/mL. Meanwhile, there is no current quality standard for FP24. In our study, the most variable affective factor during WB storage was FVIII, as might be predicted. Compared to FFP, the FVIII level decreased by 32.86% in II-FP24 and by 40.71% in I-FP24. The activity levels of other coagulation factors in I-FP24 and II-FP24 were not significantly different. These results showed that, with the exception of FVIII, FP24 would be an acceptable product for most patients who required FFP. Kleinman et al. conducted a survey in late 2009 to gather detailed information in which 40% of the respondents chose the highest category, indicating that >75% of their plasma was supplied as FP24 [16]. Interestingly, the FV activity increased substantially during the short period of storage, which was also observed in this study and might reflect activation of the contact system during storage [17]. Alhumaidan et al. also found that plasma manufactured after a 24 hr room temperature hold contains coagulation factors comparable to FFP except for a possible reduction of up to 20% in FVIII. Other clotting factors either were unchanged or showed minimal reduction (<15%). This plasma appears suitable as a transfusable product, and extension of liquid storage to 7 days merits consideration [18]. FFP had the best results for the metabolism variables K^+^, LDH, and FHb. Compared to the other 2 groups, II-FP24 had higher K^+^, LDH, and FHb levels. This result might be explained by the RBC metabolism in WB stored overnight at 4°C and the increased osmotic fragility that resulted in RBC lysis increasing some parameters in the plasma processed from WB. The differences in several biochemical parameters between the II-FP24 and P24 groups might be caused by the holding of WB at different temperatures, which induced different levels of RBC membrane fragility and deformation properties. Comprehensively, the quality of plasma from the WB that was held overnight at 4°C and then leukodepleted is much better than that of the plasma from leukodepleted WB that was then held overnight. Thus, once filtrated, plasma should be separated as soon as possible. 

Although PCs must be prepared within 6 hrs through fresh WB processing, this process is inexpedient because of blood collection from mobile vehicles or from a wide geographic area. Thus, it is important to extend the allowable processing time for PCs. The BC removal method, which is considered as a good PC preparation method, is used in many countries. Some scientists think that overnight BCs holding benefits the quantity and quality of PCs [10]. In this study, the quantity and quality of PCs from BCs and WB that were held overnight were compared. All PCs had PLT counts well above the current acceptance level of 2.0 × 10^10^/U. In particular, the PLT counts were much higher in PC2 (25.54% higher than those in PC1 and 74.50% higher than the current acceptance level). Other studies have shown levels that were 18.60%–33.00% higher in the overnight-held PCs [19, 20]. We studied the effect of BCs holding time before the PLT preparation on the quality of PCs and found lower PLT counts with a 0 hr holding time than with 4 or 16 hr holding times. Thus, we concluded that PLT recovery was comparatively affected by immediate BCs processing. This result could be explained by the relatively short rest period of BCs (i.e., the PLTs were still forming aggregates that were easily removed during centrifugation prior to disaggregation). Some biochemical indicators were different between the PC1 and PC2 groups, which could be explained by RBC metabolism in WB during overnight storage and by the lower amount of PLT in the storage bag, thus resulting in a lower total metabolism level in the bag. Although the CD62P expression was significantly different, the levels were still lower [21]. The PLT quantity is actually improved by overnight room temperature hold, and the quality is not affected.

Recently, in a special supplement report to the journal of Transfusion, scientists in 9 major blood product developmental laboratories evaluated the quality of components from WB that was held overnight at room temperature, including RBCs, plasma, and PCs. The scientists concluded that overnight holding of WB before processing had no lasting deleterious effects on the in vitro qualities of the subsequently prepared components and that using different RBC additive solutions did not appear to offer significant advantages in terms of final RBC quality, regardless of the processing method [21, 22]. Furthermore, Dijkstra-Tiekstra and colleagues analyzed the differences between PCs from fresh and overnight-stored blood. Their data showed that PCs are best prepared after a 20 to 24 hr WB hold [23]. Some scientists confirmed that storing WB for 22 ± 2 hrs at 22°C before preparing the PCs could increase the yield of PLTs in a concentrate by 43% while concurrently improving all measures of 7-day poststorage PLT viability [24, 25]. Furthermore, Cardigan and his colleague, who have extensively studied plasma, tested the coagulation factor content of plasma from WB that was stored for 24 hrs at an ambient temperature and indicated that WB storage at ambient temperature for 24 hrs had minimal effects on the plasma coagulation activity and, thus, was an acceptable alternative to producing plasma on the day of blood collection [26]. There are numerous operational and economic advantages of the blood services of ambient WB storage for 24 hrs because of the removal of the requirement to process blood for PLTs or plasma within 6 hrs of collection. Even the partial loss of FVIII in the plasma can be replaced with a biological FVIII product for clinical use when necessary.

## 5. Conclusion 

In this study in which 400 mL WB samples were divided equally, the group data revealed real differences. Overnight WB holding might be a suitable method for blood component processing. FP24 is best prepared from WB that was stored overnight at 4°C, followed by leukodepletion and separation within 24 hrs. PCs are best prepared within 6 hrs from BC that was derived from WB that was held overnight at room temperature.

## Figures and Tables

**Table 1:** Clotting factor levels and some biochemical parameters of plasma prepared from WB stored under different conditions (mean ± SD, median, and range).

	WB stored at 4°C			WB stored at 22°C	

WB hold time/components preparation (hrs)	<6/ <6	<6/18–24	18*∼*24/18–24	<6	18–24

Plasma	FFP, *n* = 12	I-FP24, *n* = 12	II-FP24, *n* = 24	P6, *n* = 12	P24, *n* = 12

Fib (g/L)	2.08 ± 0.16 2.04 (1.88– 2.26)	1.61 ± 0.43 1.48 (1.18–2.14)	2.04 ± 0.40 2.15 (1.34–2.43)	1.78 ± 0.59 1.97 (1.55–2.24)	1.79 ± 0.59 2.01 (1.52–2.19)

FV (%)	86.57 ± 18.73 92.39 (61.25–107.36)	61.14 ± 13.76* 60.64 (44.98–81.61)	75.66 ± 6.07 78.65 (67.16–81.61)	81.21 ± 6.54 78.65 (73.78–92.39)	87.59 ± 8.84 83.96 (75.78–104.14)

FVII (%)	91.71 ± 25.53 92.58 (60.52–113.00)	61.55 ± 11.45* 61.50 (44.53–75.19)	78.03 ± 19.08 71.96 (49.45–108.49)	80.32 ± 17.37 77.98 (50.92–115.29)	80.84 ± 17.29 81.02 (48.71–108.49)

FVIII (%)	97.65 ± 25.99 107.60 (63.81–128.34)	57.90 ± 7.35* 55.80 (51.26–70.31)	65.56 ± 13.93^▲^ 61.37 (45.48–92.65)	77.98 ± 11.60 78.59 (48.95–92.65)	66.30 ± 10.16^△^ 65.06 (43.88–84.37)

TP (g/L)	57.24 ± 0.81 57.40 (56.30– 58.10)	56.80 ± 4.50 57.30 (51.80–61.80)	58.96 ± 3.17 59.00 (53.90–63.90)	58.91 ± 2.94 59.10 (54.80–66.40)	59.78 ± 3.14 59.10 (56.00–66.60)

pH	7.21 ± 0.10 7.17 (7.12–7.38)	7.22 ± 0.09 7.20 (7.11–7.34)	7.24 ± 0.06 7.25 (7.17–7.36)	7.36 ± 0.07 7.35 (7.30–7.53)	7.18 ± 0.04^△^ 7.18 (7.12–7.24)

Na^+^ (mmol/L)	156.78 ± 0.88 157.00 (155.50–157.60)	154.94 ± 3.81 154.00 (151.40–159.00)	156.61 ± 2.01 156.35 (153.80–161.20)	156.05 ± 2.77 156.20 (149.70–160.00)	159.25 ± 3.24^△^ 159.20 (153.10–163.50)

K (mmol/L)	3.30 ± 0.21 3.35 (3.02–3.53)	3.96 ± 0.22* 4.03 (3.61–4.20)	4.33 ± 0.28^▲●^ 4.39 (3.71–4.69)	3.45 ± 0.21 3.44 (3.19–3.84)	4.25 ± 0.27^△^ 4.28 (3.67–4.69)

Cl^−^ (mmol/L)	70.26 ± 2.59 69.90 (67.30–73.20)	70.32 ± 3.72 70.20 (65.80–74.50)	70.92 ± 3.21 71.10 (64.80–75.70)	71.93 ± 1.95 71.95 (68.50–74.80)	69.05 ± 2.23^△^ 70.20 (65.10–72.20)

LDH (IU/L)	105.75 ± 20.56 91.00 (90.00–134.00)	100 ± 6.78 96.00 (94.00–110.00)	145.70 ± 26.00^▲●^ 151.00 (110.00–175.00)	103.58 ± 22.07 99.50 (67.00–150.00)	120.83 ± 24.39^★^ 108.00 (81.00–161.00)

GLU (mmol/L)	24.18 ± 1.24 24.58 (22.11–25.39)	22.78 ± 0.61 22.55 (22.12–23.71)	22.72 ± 1.18 22.92 (21.21–24.38)	23.09 ± 1.38 22.78 (21.02–25.48)	21.93 ± 1.48 21.79 (19.89–25.21)

FHb (mg/L)	16.34 ± 7.88 10.02 (1.23–29.91)	43.54 ± 19.76* 44.39 (20.81–73.03)	129.96 ± 34.33^▲●^ 129.94 (71.02–177.17)	22.15 ± 19.51 13.71 (1.67–66.56)	27.46 ± 29.11^★^ 14.40 (3.89–89.74)

*P < 0.05 versus FFP; ^▲^ *P* < 0.05 versus FFP.

^△^ *P* < 0.05 versus P6; ^★^ *P  *<  0.05 versus II-FP24.

^●^ *P* < 0.05 versus I-FP24.

**Table 2:** In vitro quality variables of PCs collected under various conditions (mean ± SD, median, and range).

	PC1 (*n* = 12)	PC2 (*n* = 12)
PLTs (×10^10^/U)	2.78 ± 1.49 2.6 (1.11–6.58)	3.49 ± 1.19 3.33 (2.11–5.60)
pH	7.12 ± 0.05 7.13 (7.03–7.17)	6.90 ± 0.04* 6.89 (6.84–6.99)
pCO_2_(mmHg)	34.61 ± 3.01 35.45 (29.80–39.20)	49.19 ± 5.07* 47.90 (43.80–57.50)
pO_2 _(mmHg)	117.50 ± 10.46 113.00 (101.00–135.00)	94.81 ± 17.50* 95.40 (60.90–117.00)
K^+^ (mmol/L)	3.01 ± 0.21 2.95 (2.70–3.30)	3.33 ± 0.24* 3.40 (3.00–3.80)
Na^+^ (mmol/L)	134.73 ± 1.56 135.00 (131.00–137.00)	131.36 ± 2.11* 132.00 (129.00–135.00)
Cl^−^ (mmol/L)	73.00 ± 2.05 73.00 (69.00–76.00)	73.00 ± 1.73 72.00 (71.00–76.00)
GLU (mmol/L)	19.17 ± 1.13 19.00 (17.80–21.40)	20.14 ± 1.28 20.20 (18.20–22.60)
Lac (mmol/L)	3.25 ± 0.70 3.10 (2.30–4.70)	4.13 ± 0.41* 4.20 (3.60–4.70)
CD62P expression (%)	10.06 ± 0.28 10.06 (9.86–10.25)	11.93 ± 0.18* 11.93 (11.80–12.06)
Adhesion (%)	43.21 ± 2.27 43.21 (41.61–44.81)	40.37 ± 3.66 40.37 (37.78–42.95)
HSR%	1.01 ± 2.68	
Bacterial culture	Neg	Neg

*P < 0.05 versus PC1.

## Abbreviations

FFP: Fresh frozen plasma
FP24: Plasma frozen within 24 hrs of phlebotomy
BC(s): Buffy coat(s)
HSR: Hypotonic shock response
PC(s): Platelet concentrate(s)
PLT: Platelet
WB: Whole blood
LDH: Lactate dehydrogenase
FHb: Free hemoglobin
hrs: Hours
TP: Total protein
APTT: Activated partial thromboplastin time
PT: Prothrombin time
GLU: Glucose
Lac: Lactic acid.
